# Highly Unsaturated Platinum and Palladium Carbenes PtC_3_ and PdC_3_ Isolated and Characterized in the Gas Phase

**DOI:** 10.1002/anie.201511646

**Published:** 2016-02-16

**Authors:** Dror M. Bittner, Daniel P. Zaleski, David P. Tew, Nicholas R. Walker, Anthony C. Legon

**Affiliations:** ^1^ School of Chemistry, Bedson BuildingNewcastle UniversityNewcastle upon Tyne, Tyne and WearNE1 7RUUK; ^2^ School of ChemistryUniversity of BristolBristolBS8 1TSUK; ^3^ Argonne National Laboratory, Chemical Sciences and Engineering9700 S. Cass Ave., Bldg. 200LemontIL60439USA

**Keywords:** ab initio calculations, carbenes, palladium, platinum, rotational spectroscopy

## Abstract

Carbenes of platinum and palladium, PtC_3_ and PdC_3_, were generated in the gas phase through laser vaporization of a metal target in the presence of a low concentration of a hydrocarbon precursor undergoing supersonic expansion. Rotational spectroscopy and ab initio calculations confirm that both molecules are linear. The geometry of PtC_3_ was accurately determined by fitting to the experimental moments of inertia of twenty‐six isotopologues. The results are consistent with the proposal of an autogenic isolobal relationship between O, Au^+^, and Pt atoms.

The importance of industrial catalysis by platinum and palladium has prompted extensive studies of their gas‐phase chemistry.^1^ Each metal atom is known to initiate cleavage of the C−H and C=C bonds of hydrocarbon precursors. We believe that the present study provides the first pure rotational spectra of platinum and palladium carbenes isolated in the gaseous phase. PtC_3_ and PdC_3_ (each in a ^1^Σ state) were generated through laser vaporization of solid Pt/Pd in the presence of a gas sample undergoing supersonic expansion and containing a low concentration (typically 1 %) of a hydrocarbon precursor in a buffer gas of argon. Analysis of the rotational spectra reveals that each molecule has a linear geometry and an MCCC connectivity (where M is the metal atom). The results are a successful test of a model proposed by Pyykkö et al.^2^ which suggests that platinum can be regarded as the isoelectronic and isolobal counterpart of a chalcogen for the purposes of predicting structure and reactivity trends.

A wide range of hydrocarbon precursors, each tested individually, were found to allow the generation of PtC_3_ and PdC_3_. For PdC_3_, the range of effective precursors includes C_3_H_4_ (allene), C_2_H_2_, C_2_H_4_, CH_4_, and C_4_H_4_O (furan). For PtC_3_ the range is narrower, including C_3_H_4_ (allene), C_2_H_4_, and CH_4_, all of which were found to be effective. Broadband microwave spectra of the target molecules were recorded between 6.5 and 18.5 GHz (Figure 1) using a spectrometer described previously in detail.^3^ Each spectrum was assigned and fitted to the Hamiltonian of a linear molecule using Western's program PGOPHER.^4^ The low number of *J*′→*J*′′ transitions within the bandwidth of the spectrometer required that centrifugal distortion constants be fixed at results calculated ab initio by an approach described previously.^5^ Structure optimizations, reaction energies, and orbital energy level diagrams were calculated using the MOLPRO package^6^ at the CCSD(T) level of theory.^7^ The basis set combination employed the aug‐cc‐pwCV5Z basis set for each C atom and the aug‐cc‐pwCV5Z‐PP basis set for each of Pt and Pd.^8^ The ECP‐28‐MDF and ECP‐60‐MDF effective core potentials were used to account for scalar relativistic effects on Pd and Pt, respectively,^8^ with all electrons included in the correlation treatment. Electric dipole moments and centrifugal distortion constants were calculated with the GAUSSIAN 09 package^9^ at the MP2 level of theory using a basis set combination consisting of aug‐cc‐pVTZ on C atoms and aug‐cc‐pVTZ‐PP on Pd and Pt atoms.^8^ Selected results of spectroscopic fits are shown in Table 1 with complete details for all isotopologues provided in the Supporting Information. The standard deviations of all fits are consistent with the measured linewidth (FWHM) of 120 kHz. Neither PtC_2_ nor PdC_2_ were identified despite a careful search of the spectra. Rotational transitions of both PtC^10^ (measured previously) and PdC lie higher in frequency than the upper limit of the spectrometer. Where PdC_3_ was generated from a furan precursor, intense transitions of PdCO^11^ were detected in addition to those assigned to PdC_3_.

**Figure 1:**
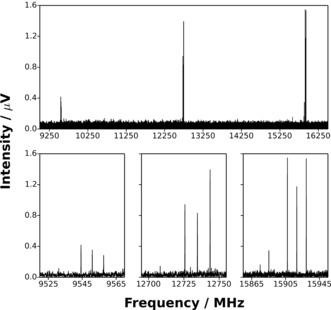
Top: The broadband rotational spectrum (showing the 9 GHz to 16.5 GHz region) averaged over 480 000 free induction decays (FIDs) and recorded while vaporizing a Pt source in the presence of CH_4_ under the conditions described in the text. Bottom: *J*′→*J*′′=6→5 transitions of each of ^194^PtC_3_, ^195^PtC_3_, ^196^PtC_3_, and ^198^PtC_3_ are displayed in the bottom‐left panel. *J*′→*J*′′=7→6 and *J*′→*J*′′=8→7 transitions of the isotopologues listed above are shown in the bottom‐center and bottom‐right panels, respectively.

**Table 1:** Spectroscopic parameters of PtC_3_ and PdC_3_.^[a]^

Species	*B*_0_ [MHz]	[*D_J_*×10^2^]^[b]^ [kHz]	*χ_aa_*(^105^Pd) [MHz]	*σ* [kHz]	*N*
^194^Pt^12^C^12^C^12^C	1592.94589(35)	[6.2]	–	5.0	3
^194^Pt^13^C^13^C^13^C	1486.74426(60)	[5.4]	–	11.1	4
^194^Pt^12^C^12^C^13^C	1522.47541(52)	[5.6]	–	9.1	3
^194^Pt^12^C^13^C^12^C	1560.77071(27)	[6.0]	–	3.4	2
^194^Pt^13^C^12^C^12^C	1585.00114(43)	[6.2]	–	6.1	3
					
^106^Pd^12^C^12^C^12^C	1702.33446(43)	[9.5]	–	6.3	4
^105^Pd^12^C^12^C^12^C	1705.89034(57)	[9.6]	35.88(43)	14.5	9
^106^Pd^13^C^13^C^13^C	1599.5465^[c]^	[8.4]	–	–	1

[a] Results of selected spectroscopic fits illustrating the dependence of rotational (*B*_0_) and centrifugal distortion (*D_J_*) constants on isotopic substitution. *χ_aa_*(^105^Pd) denotes the nuclear quadrupole coupling constant of the ^105^Pd atom. *N* and *σ* are the number of fitted transitions and the standard deviation of the fit, respectively. Further results are presented in Table S1 in the Supporting Information. [b] Centrifugal distortion constants are fixed to results calculated ab initio at the MP2/AVTZ level. [c] Result calculated from a single transition frequency.

Spectra were measured for isotopologues of PtC_3_ and PdC_3_ that contain the ^13^C isotope to ensure assignment of the correct molecular carriers and allow precise determination of the molecular geometries. Experimental data are available only for the ground vibrational state of each molecule allowing an effective *r*_0_ geometry to be fitted in each case. The experimental results are consistent with two possibilities for each molecule: 1) a geometry that is slightly bent at equilibrium but quasilinear in the *v*=0 state, and 2) an equilibrium (*r*_e_) geometry that is linear. The ab initio calculations suggest that both molecules are linear at equilibrium. The intensities of PdC_3_ transitions were found to be highly dependent on the choice of precursor, in the order C_3_H_4_>C_2_H_4_>CH_4_. Transition intensities were lower when the population of PdC_3_ was divided across many isotopic permutations and isotopically enriched allene is prohibitively expensive. These factors prevented measurement of the spectrum of any PdC_3_ isotopologue that contains both ^12^C and ^13^C isotopes. The intensities of PtC_3_ transitions were insensitive to the choice of precursor and it was possible to generate and record spectra for many isotopic permutations of PtC_3_ (from the set of ^194^Pt, ^195^Pt, ^196^Pt, ^198^Pt, ^12^C, and ^13^C atoms) using samples prepared by mixing ^12^CH_4_ and commercially supplied ^13^CH_4_. It was also found that PtC_3_ can be generated from a mixture of ^12^C_2_H_2_ and ^13^CH_4_ precursors with the result that the spectra of ^194^Pt^12^C^12^C^13^C, ^194^Pt^12^C^13^C^12^C, and ^194^Pt^13^C^12^C^12^C were detected with equal intensities. The observation that the ^13^C isotope does not preferentially occupy an end position of the C_3_ subunit strongly implies that the C≡C bond of C_2_H_2_ cleaves during the sequence of reactions that generates PtC_3_ from this set of precursors.

The present study is believed to be the first to characterize MC_3_ units by rotational spectroscopy. Transition‐metal dicarbides, such as ScC_2_ and YC_2_, have been studied previously.^12^ The dipole moments of PdC_3_ and PtC_3_ are calculated at the MP2 level to be 6.1 and 5.6 D, respectively. The lengths of bonds within PtC_3_ were fitted to experimentally determined rotational constants using Kisiel's STRFIT.^13^ Spectra were measured for 26 distinct isotopologues of PtC_3_ where the set includes every permutation of C_3_ that it is possible to generate from ^12^C and ^13^C isotopes. The bond lengths thus determined are compared with those in isolated PtC, C_3_ and OC_3_ molecules in Table 2. The *r*_0_ geometry of PtC_3_ is in good agreement with the *r*_e_ geometry calculated at the CCSD(T) level. The Pt−C bond in PtC_3_ is longer than found in diatomic PtC^10^ by 0.053 Å. There are similarities between *r*(MC) in PtC_3_ and in PtCO,^14^ and also in changes when these molecules form from their component Pt and C_3_/CO subunits. The *r*(MC) parameter in PtC_3_ is shorter than the same quantity in PtCO by 0.031 Å. The first C=C bond (that which is contiguous with the Pt−C bond) of PtC_3_ is longer than the C=C bond in isolated C_3_ by 0.022 Å. The set of isotopologues studied is less extensive for PdC_3_ than for PtC_3_ and does not permit determination of all bond lengths from the experimental data. If the lengths of C=C bonds within the molecule are fixed as shown in Table 2, *r*(PdC) is determined to be 1.79898(4) Å. Values of vibrational wavenumbers calculated ab initio are provided in the Supporting Information.

**Table 2:** Structural parameters of PtC_3_, PdC_3_, and related molecules.^[a]^

Species^[a]^	*r*(MC) [Å]	*r*(CC1) [Å]	*r*(CC2) [Å]
PtC^[b]^	1.679	–	–
C_3_ (*r*_0_)^[b]^	–	1.277247(2)	1.277247(2)
OC_3_ (*r*_0_)^[b]^	1.150	1.306	1.254
PtC_3_ (*r*_0_)	1.7315(14)	1.2993(19)	1.2759(11)
PtC_3_ (*r*_e_)	1.7280	1.2942	1.2836
PdC_3_ (*r*_0_)	1.79898(4)	[1.3009]^[c]^	[1.2789]^[c]^
PdC_3_ (*r*_e_)	1.7962	1.2958	1.2866

	*r*(MC) [Å]	*r*(CO) [Å]	
CO	–	1.128	
PtCO (*r*_0_)^[d]^	1.7625(4)	1.1466(6)	
PdCO (*r*_0_)^[d]^	1.8447(1)	1.1374(2)	

[a] *r*(MC) denotes the bond between the metal atom (or oxygen atom in OC_3_) and its coordinated carbon. *r*(CC1) denotes the C=C bond nearest to the metal atom with *r*(CC2) used to label the other. *r*_0_ values are determined experimentally and *r*_e_ values are calculated ab initio. [b] Data from Refs. 10, 19, 22. [c] Each number in square brackets is fixed to the result obtained by correcting the *r*_e_ value calculated ab initio for PdC_3_ for the difference between the *r*_0_ and *r*_e_ values determined for the equivalent parameter in PtC_3_. [d] Data from Refs. 11, 14.

The described results confirm that the heavier elements of Group 10 can form linear arrangements similar to that previously identified for Ni_2_C_3_.^15^ The detected palladium/platinum carbenes are amongst the smallest to be structurally characterized.^16^ There is a correspondence between the linear geometries of the MC_3_ units identified herein and the linear carbon chains that are interceded by Pt/Pd atoms which are a feature of many synthetic coordination polymers.^17^ The results are also interesting in the context of the wider chemistry of metal atoms in hydrocarbon plasmas. Early transition metals are known to react with hydrocarbon precursors to generate metallocarbohedrynes (met‐cars).^18^ Late transition metals show no general tendency to form such extended structures. The present experiment does not unambiguously distinguish the reaction sequences (or networks of competing reactions) that generate PdC_3_ and PtC_3_. It is possible that a fraction of the population of each forms through gas‐phase association of individual metal atoms with intact C_3_ or other units generated independently of any metal atom.^19^ The energy changes accompanying the M+C_3_→MC_3_ association reactions to yield linear MC_3_ units are calculated to be −295 kJ mol^−1^ and −417 kJ mol^−1^ when M=Pd and M=Pt, respectively (detailed calculations are shown in the Supporting Information). However, it is also possible that the metals themselves initiate the sequence of chemical reactions that leads to dehydrogenation of the precursor. There is extensive evidence from previous studies that both Pt and Pd atoms undergo bond‐insertion and cleavage reactions with hydrocarbons.1a, 20 MCH_2_ and MCCH_2_ have both been generated1a,1b, 21 previously by a laser vaporization/supersonic expansion method, characterized by matrix isolation spectroscopy, and are also likely to be generated under the present experimental conditions. Transition frequencies of MCH_2_ are expected to be above the upper frequency limit of the spectrometer and both MCH_2_ and MCCH_2_ will have comparatively low dipole moments which significantly decrease the intensity of their rotational transitions relative to those of MC_3_.

An empirical model proposed by Pyykkö et al.2a provides a chemical rationalization for an enhanced stability of MC_3_ relative to MC_2_ or MC_4_. Calculations of the geometries of CAu^2+^, CAu^3+^, Pt_2_C, Pt_2_C_3_, and Au_2_C_2_ revealed analogies between the behavior of each of Au^+^ and Pt and a chalcogen atom such as O.2a Within this model, the σ hole on platinum arising from the 5d^10^6s^0^ configuration is analogous to the 2pσ^0^ hole on oxygen, and the 5dπ orbitals of platinum participate in π‐bonding interactions analogous to those involving the 2pπ orbital of oxygen. The existence of a family of stable molecules was thus predicted. An orbital energy level diagram for PtC_3_ is presented in Figure S1 in the Supporting Information. There are striking similarities between the geometries of MC_3_ measured during the present work and that reported earlier for OC_3_ by Brown et al.^22^ Applying the model of Pyykkö et al., PtCO, PtC, and Pt_2_C_3_ are analogues of the well‐known, stable oxocarbons carbon dioxide, monoxide, and suboxide, respectively, each of which have been known since the 19th century. Similarly, PtSi^23^ can be regarded as an analogue of SiO. The oxocarbon analogue of Pt_2_C_2_ would be ethylene dione,^24^ a transient species characterized, only through spectroscopy, for the first time in 2015. This model can thus explain why PtCO, PtC, and PtC_3_, but not yet PtC_2_, have been detected. The results of the present work thus support the suggestion that platinum can be regarded an isoelectronic, isolobal counterpart of oxygen. The proposal can be further assessed with reference to previous works.

Reports of clusters containing multiple carbon and platinum or palladium atoms are scarce. The adsorption of, and reactions of, CH_4_ and CO on Pt_*n*_ clusters1e and the structures of Pt_*n*_O_*m*_ clusters1d have been studied. Harding et al. identified a Pt_3_C^+^ cluster ion^25^ for which the geometry is analogous to a carbonate ion and hence consistent with the prediction of the model provided by Pyykkö et al.^2^ The geometries of other platinum/carbon clusters, which have not yet been observed or characterized, may perhaps be predicted by analogy with other oxocarbons. For example, mellitic anhydride (C_12_O_9_) is known to be stable, suggesting that Pt_9_C_12_ might be generated in an equivalent structural form. An experimental study^26^ of AuC_*n*_^+^ and CuC_*n*_^+^ revealed ion intensities in the mass spectra that are significantly stronger where *n*=3 than for clusters of other sizes.^26^ Some caution must be exercised in drawing conclusions about the thermodynamic stability of AuC_3_^+^ relative to other cluster sizes from these results. As in the present work, the experiment performed by Ticknor et al.^26^ did not unambiguously distinguish between various factors that contribute to observed spectral intensities. It is likely that C_3_ was generated with a significantly higher abundance^19^ than C_2_ within the expanding gas sample and this may cause the generation of AuC_3_^+^ to be favored over the generation of clusters of other sizes, regardless of the thermodynamic stability of AuC_3_^+^. Indeed, during a previous study, signals for NiC_3_^+^ and NiC_6_^+^ were detected in mass spectra with higher intensity than units containing 1, 2, 4, or 5 carbon atoms,^27^ although the Ni^+^ ion is not isoelectronic and isolobal with O. However, the reported fragmentation behavior of AuC_*n*_^+^ is also notable. Clusters where *n* is odd lose only the metal atom on photodissociation whereas those with an even value of *n* display an additional loss channel corresponding to the loss of an odd number of carbon atoms. The overall result is that chains (either isolated or attached to the metal ion) containing an odd number of carbon atoms tend to be formed during photofragmentation, consistent with the proposal of Pyykkö et al. The perspective thus emerging from the collected results of spectroscopic experiments is that the proposal2a of an autogenic isolobal relationship of Pt and Au^+^ centers with the O atom is powerful and useful with respect to structural trends in gas‐phase clusters that contain Pt, Pd, Au^+^, and C centers.

## Supporting information

As a service to our authors and readers, this journal provides supporting information supplied by the authors. Such materials are peer reviewed and may be re‐organized for online delivery, but are not copy‐edited or typeset. Technical support issues arising from supporting information (other than missing files) should be addressed to the authors.

Supplementary
